# Academic stressors and coping strategies among MBBS students: a cross-sectional, year-wise comparative study

**DOI:** 10.3389/fmed.2026.1789919

**Published:** 2026-03-04

**Authors:** Priyanka Rathi, Keshav Banga, Himanshu Singh

**Affiliations:** National Institute of Medical Sciences and Research, Nims University Rajasthan, Jaipur, Rajasthan, India

**Keywords:** academic stress, coping behavior, cross-sectional studies, medical education, MSSQ

## Abstract

**Background:**

Medical students experience high levels of stress that vary across different phases of training. Identifying specific stressors and the ways students cope with them is essential for designing meaningful and targeted interventions.

**Objective:**

To assess and compare stressors and coping mechanisms among MBBS students across different academic years.

**Methods:**

A cross-sectional, questionnaire-based study was conducted among MBBS students and interns at a tertiary medical institute using the Medical Student Stressor Questionnaire (MSSQ) and Brief COPE. Domain-wise stress severity and coping strategies were analyzed. Inferential statistics included Kruskal–Wallis tests with post-hoc comparisons, chi-square analysis for stress severity distribution, and Spearman correlation to examine associations between stress and coping.

**Results:**

Academic-related stress was the only stress domain that differed significantly across academic years (H = 48.72, *p* < 0.001), with final-year students reporting the highest stress levels. Distribution of stress severity differed significantly across domains (χ^2^ = 364.60, *p* < 0.001), with academic stress accounting for a greater proportion of high and severe stress categories. Problem-focused coping showed the highest overall mean scores, but coping strategies did not significantly differ across academic years. Weak positive correlations were observed between overall stress levels and problem-focused (*ρ* = 0.232), emotion-focused (ρ = 0.266), and avoidant coping (ρ = 0.277) (all *p* < 0.001).

**Conclusion:**

Academic-related stress represents the primary domain showing meaningful variation across stages of medical training, particularly in final-year students. Coping patterns appear relatively stable across academic years, with higher stress associated with increased use of coping behaviors overall. These findings provide a statistically grounded basis for future longitudinal research and targeted strategies addressing academic stress in medical education.

## Introduction

Medical education is often considered one of the most demanding forms of professional training, owing to intensive academic workloads, frequent assessments, and early clinical responsibilities (1, 2). Medical students are expected to acquire extensive theoretical knowledge while simultaneously developing clinical competence, professionalism, and emotional resilience. Consequently, stress has become a pervasive phenomenon among medical students globally, with prevalence rates consistently higher than those observed in age-matched peers from non-medical disciplines (3).

Sustained exposure to stress during undergraduate medical training has been linked to a broad range of adverse outcomes, including anxiety, depression, burnout, impaired academic performance, reduced empathy, and diminished quality of life (4–6). Evidence further suggests that psychological distress during medical college may persist into residency and professional practice, adversely affecting physician well-being, patient care, and healthcare systems (7). When stress remains unrecognized or inadequately managed, students may adopt maladaptive coping strategies such as avoidance, emotional disengagement, excessive digital media use, alcohol consumption, smoking, or other substance-related behaviors (8, 9).

The nature and intensity of stressors encountered by medical students are not static but vary across different phases of training. Early academic years are often dominated by adjustment challenges, heavy theoretical curricula, fear of failure, and competitive learning environments. In contrast, senior students face additional stressors related to clinical responsibilities, patient care, major professional examinations, and uncertainty regarding postgraduate training and career prospects (10, 11). Despite this dynamic progression, much of the existing literature examines stress among medical students as a uniform construct, with limited emphasis on comparative, year-wise analysis of stressors and coping mechanisms (12).

Coping strategies play a pivotal role in modulating the psychological impact of stress. Adaptive coping mechanisms—such as problem-focused coping, seeking social support, physical activity, mindfulness, and cognitive reframing—have been shown to protect against emotional exhaustion and burnout (13). Conversely, maladaptive coping strategies are associated with higher psychological morbidity, academic disengagement, and increased vulnerability to addiction and other harmful behaviors (9, 14). A nuanced understanding of how coping strategies interact with specific stressors across academic years is therefore essential for designing targeted and effective interventions.

Although awareness about mental health in medical education has increased, existing support systems are still underutilized, often due to stigma and practical barriers (15). Emerging approaches in mental health support and digital well-being tools may offer future opportunities for enhancing stress monitoring and student support systems. However, their potential role within medical education warrants further empirical investigation before implementation-oriented conclusions can be drawn.

Against this background, the present study aimed to conduct a comparative analysis of stressors and coping mechanisms among MBBS students across different academic years. By identifying key stress domains and associated coping patterns, this research seeks to generate evidence-based insights to inform targeted institutional interventions.

## Methodology

This cross-sectional, questionnaire-based study was conducted in accordance with the Strengthening the Reporting of Observational Studies in Epidemiology (STROBE) guidelines for cross-sectional research. The study has been approved by the Institutional Ethics Committee (IEC) of NIms University (Letter No. EC/NEW/INST/2022/RJ/0118).

### Participants

The study population comprised undergraduate medical students across all academic years including interns enrolled at the National Institute of Medical Sciences & Research (NIMS&R), Jaipur. Data collection was carried out over a three-month period (January–March 2025). A convenience sampling approach was adopted due to logistical feasibility; however, efforts were made to ensure representation across all academic years to enable comparative analyses. Participation was voluntary and anonymous, and no incentives were provided. Students willing to participate voluntarily were enrolled while students with self-reported pre-existing psychiatric illness were excluded from the study to minimize potential confounding effects on stress perception and coping behavior. A written informed consent was taken from all participants.

A total of 311 MBBS students and interns participated in the study. Participants represented all academic years, with the largest proportion from second year (*n* = 134, 43.1%), followed by third year (*n* = 88, 28.3%), final year (*n* = 69, 22.2%), first year (*n* = 16, 5.1%), and internship (*n* = 4, 1.3%). The sample included 164 females (52.7%) and 147 males (47.3%). Most participants were hostellers (*n* = 300, 96.5%), while a small proportion were day scholars (*n* = 11, 3.5%). Regarding examination exposure, 187 participants (60.1%) reported having appeared for an examination within the previous month, and 297 participants (95.5%) indicated that they had an upcoming examination within 1 month, reflecting a predominantly exam-oriented academic context during the period of data collection.

### Measures

Data were collected using a structured, pre-validated, self-administered questionnaire incorporating items from two validated instruments: the Medical Student Stressor Questionnaire (MSSQ) (16) and the Brief COPE inventory (17), both commonly used in medical education research to assess stress and coping behavior. Stressors were assessed using the Medical Student Stressor Questionnaire (MSSQ), a validated instrument specifically developed to identify domain-specific sources of stress among medical students. The MSSQ has demonstrated acceptable psychometric properties across medical student populations and is widely used in academic stress research. Coping strategies were assessed using the Brief COPE inventory, a multidimensional and psychometrically validated measure of coping responses. The Brief COPE has been extensively applied in health and educational research and is not norm-referenced; instead, it is intended to characterize relative patterns of coping behavior within a study population.

The questionnaire consisted of three sections:

The first section collected demographic and contextual information including academic year, gender, hosteller/day scholar, recent/upcoming exams.
The second section was on assessment of stressors (MSSQ-based domains): Stressors were categorized into six domains (Academic stressors, Interpersonal and intrapersonal stressors, Teaching-learning related stressors, Social stressors, Drive & desire-related stressors, Group activity-related stressors). Participants rated each item on a 5-point Likert scale ranging from 0 (no stress) to 4 (severe stress). Domain-level mean scores were calculated for analysis.
Stress severity categories were interpreted using established MSSQ scoring ranges: 0–1.00 - Mild; 1.01–2.00 - Moderate; 2.01–3.00 - High; 3.01–4.00 - Severe Stress. These classifications provide a standardized method for stratifying perceived stress severity across domains.
The third section was on assessment of coping mechanisms (brief COPE-based): Coping strategies were grouped into three categories: problem-focused coping, emotion-focused coping, and avoidant coping strategies. Responses were rated on a 4-point Likert scale ranging from 1 (not at all) to 4 (a lot), reflecting the frequency of use of each coping behavior. Mean scores were calculated for each coping category.

### Procedure

Participants were approached during lecture sessions, hostel accommodations, and clinical postings. The questionnaire was administered electronically using Google Forms allowing anonymous self-completion and minimizing interviewer influence. The form was open to accept responses for 1 month. Reporting of this online survey was guided by the Checklist for Reporting Results of Internet E-Surveys (CHERRIES). Completed responses were automatically captured and exported into Microsoft Excel for further analysis. All responses were screened for completeness and consistency prior to analysis. Duplicate entries and incomplete questionnaires were excluded. A total of 600 MBBS students and interns were invited to participate in the online survey using Google Form during the study period. Of these, 360 responses were received. After screening for completeness and eligibility, 49 responses were excluded due to incomplete submissions, duplicate entries, or failure to meet inclusion criteria. The final analysis included 311 participants, representing a response rate of 51.8% of the eligible student population at the institution.

Data was coded numerically to facilitate statistical analysis. Access to the dataset was restricted to the principal investigators, and data were securely stored for research purposes only.

### Statistical analysis

Data were analyzed using SPSS version 26. Continuous variables were summarized as mean ± standard deviation, while categorical variables were presented as frequencies and percentages. Normality was assessed using the Shapiro–Wilk test. As several variables were not normally distributed, non-parametric tests were applied.

Differences in stress and coping scores across academic years were analyzed using the Kruskal–Wallis test, with Dunn-Bonferroni post-hoc comparisons performed where applicable. Associations between stress and coping scores were examined using Spearman rank correlation coefficients. Distribution of stress severity categories was assessed using Chi-square tests of independence. Effect sizes (Cramer’s V and epsilon-squared) were reported where appropriate. Statistical significance was set at *p* < 0.05.

## Results

A total of 311 MBBS students and interns voluntarily participated and completed the questionnaire, yielding complete datasets suitable for analysis. Participants represented all academic years, enabling year-wise comparisons of stress domains and coping strategies.

### Domain-wise stress scores across academic years

The domain-wise mean stress scores across academic years are presented in Table 1. The mean stress levels varied considerably across domains and academic progression. Inferential analysis using the Kruskal–Wallis test demonstrated statistically significant differences across academic years only for academic-related stress (H = 48.72, *p* < 0.001, ε^2^ = 0.146), indicating a moderate effect size. Post-hoc comparisons revealed significantly higher academic stress among final-year students compared with first-, second-, and third-year students, while second-year students also showed higher academic stress than third-year students.

**Table 1:** Domain wise mean stress score across all the academic years (*N* = 311).

Domains	First year (mean stress score)	Second year (mean stress score)	Third year (mean stress score)	Final year (mean stress score)	Internship (mean stress score)	Total (*N* = 314)
1 (Academic-related stress)	2.02	2.28	1.83	3	2.04	2.03
2 (Interpersonal and intrapersonal stress)	1.34	1.27	1.33	1.63	1.12	1.37
3 (Teaching-learning related stress)	1.31	1.07	1.04	1.37	0.75	1.41
4 (Social-related stress)	0.95	0.88	0.98	1.19	0.83	0.98
5 (Drive and desire-related stress)	0.31	1.1	0.87	1.15	1.5	1.06
6 (Group activity-related stress)	0.81	0.99	1.02	1.46	2	1.11

Interpersonal and intrapersonal stress consistently reflected moderate levels across cohorts (overall mean 1.37). Teaching–learning related stress was largely mild to moderate, lowest among interns, yielding an overall moderate mean (1.41). Social-related stress remained uniformly mild across all years (overall mean 0.98). Drive and desire-related stress showed variability, ranging from mild in early years to higher levels among interns, with an overall moderate mean (1.06). Group activity-related stress increased with academic seniority, reaching moderate levels in final-year and internship students (overall mean 1.11). No statistically significant year-wise differences were observed in interpersonal and intrapersonal stress, teaching–learning related stress, social-related stress, drive and desire-related stress, or group activity-related stress (*p* > 0.05).

**Figure 1:**
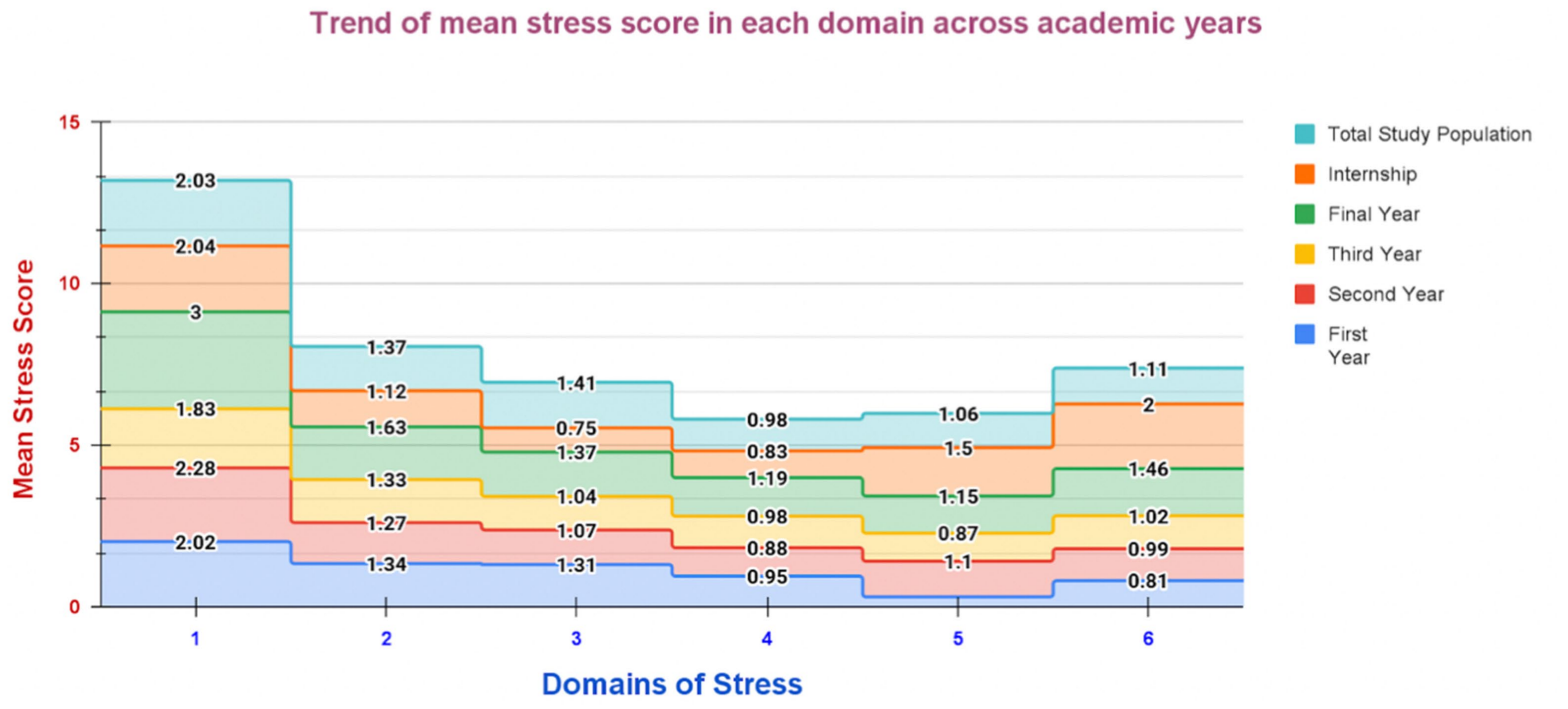
Domain-wise trends in mean stress scores across academic years among MBBS students (*N* = 311).

Overall, academic-related stress emerged as the only stress domain demonstrating meaningful variation across training phases, with peak scores observed during the final year (Figure 1).

### Distribution of stress severity across domains

**Figure 2:**
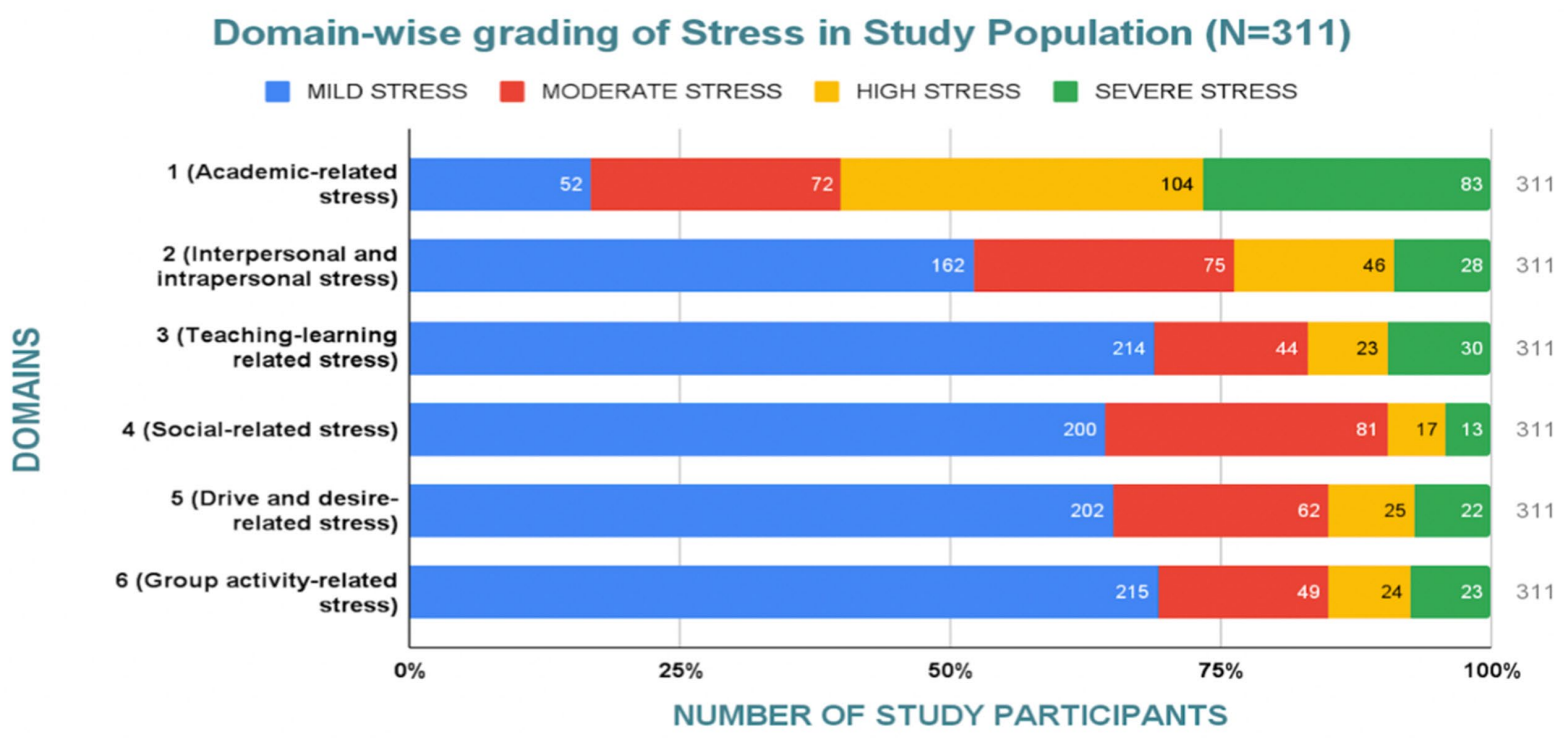
Stacked distribution of mild, moderate, high, and severe stress across six stress domains among MBBS students (*N* = 311).

The distribution of stress severity among the study population across six stress domains is summarized in Table 2 and illustrated in Figure 2.

**Table 2:** Severity grading of stress across different stress domains in the study population (*N* = 311).

Domains	Mild stress	Moderate stress	High stress	Severe stress
	*n*	*n*	*n*	*n*
1 (Academic-related stress)	52	72	104	83
2 (Interpersonal and intrapersonal stress)	162	75	46	28
3 (Teaching-learning related stress)	214	44	23	30
4 (Social-related stress)	200	81	17	13
5 (Drive and desire-related stress)	202	62	25	22
6 (Group activity-related stress)	215	49	24	23

Chi-square analysis demonstrated a significant association between stress domain and severity category (χ^2^ = 364.60, df = 15, *p* < 0.001, Cramer’s V = 0.255), indicating moderate differences in severity distribution across domains.

Academic-related stress showed a disproportionately higher proportion of high and severe stress categories compared with other domains, whereas social-related, teaching–learning, and group activity-related stress were predominantly classified as mild. These findings highlight academic stress as the principal contributor to overall stress burden among participants.

### Coping mechanisms across academic years

Table 3 summarizes the mean scores of the three coping mechanism categories—problem-focused, emotion-focused, and avoidant—across various academic years. Although problem-focused coping demonstrated the highest overall mean scores and avoidant coping the lowest, Kruskal–Wallis analysis showed no statistically significant differences across academic years for problem-focused (H = 5.27, *p* = 0.261), emotion-focused (H = 4.97, *p* = 0.291), or avoidant coping (H = 3.07, *p* = 0.547). Thus, coping strategy preferences appeared relatively stable across different stages of medical training.

**Table 3:** Coping mechanisms (mean score) across academic years.

Coping mechanism	First year (mean score)	Second year (mean score)	Third year (mean score)	Final year (mean score)	Internship (mean score)	Total (mean score)
Problem focused	2.46	2.29	2.45	2.52	2	2.39
Emotion focused	2.4	2.22	2.33	2.44	2	2.31
Avoidant	1.82	2.001	1.93	1.97	1.79	1.96

### Association between stress levels and coping strategies

**Figure 3:**
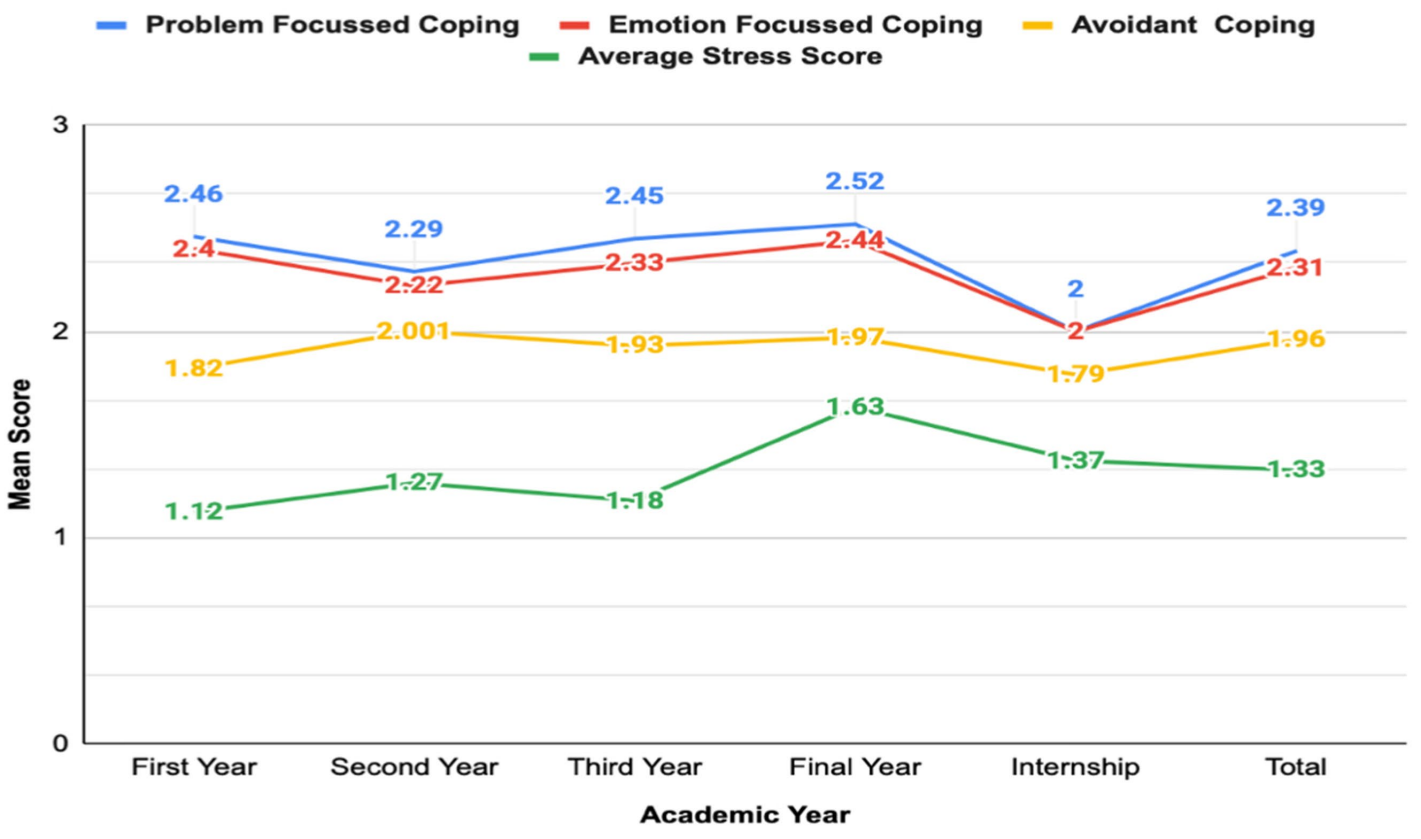
Comparison of coping mechanisms and mean stress scores across academic years among MBBS students.

Figure 3 depicts trends in mean coping mechanism scores and mean stress scores across academic years among MBBS students. Spearman rank correlation analysis revealed weak but statistically significant positive associations between overall stress and all coping mechanisms: problem-focused coping (*ρ* = 0.232, *p* < 0.001), emotion-focused coping (ρ = 0.266, *p* < 0.001), and avoidant coping (ρ = 0.277, *p* < 0.001). These findings suggest that students experiencing higher stress levels reported greater use of coping behaviors in general rather than selectively increasing adaptive coping strategies alone.

### Distribution of coping scores

**Figure 4:**
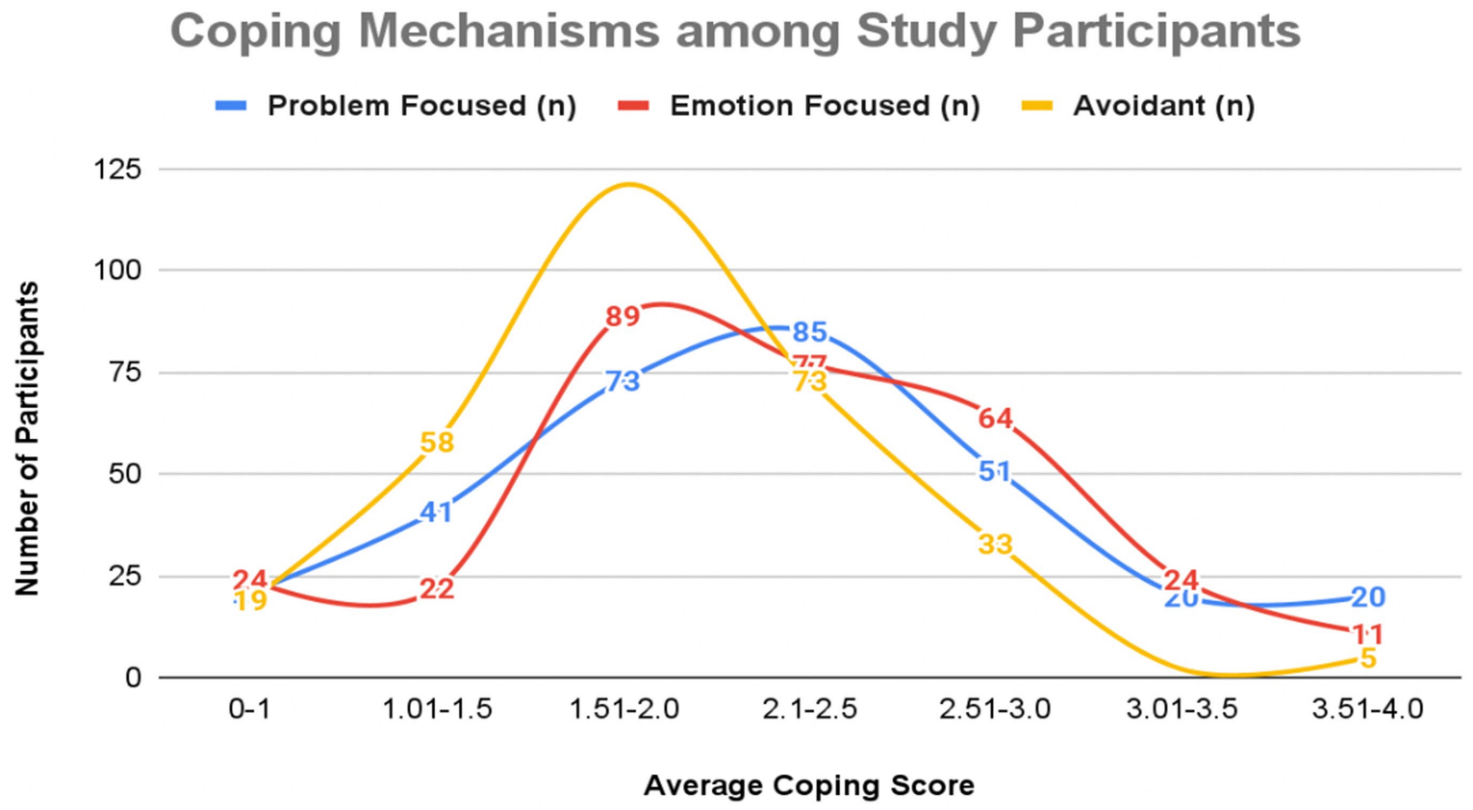
Distribution of participants according to their average coping scores.

Figure 4 illustrates the distribution of participants according to their average coping scores for problem-focused, emotion-focused, and avoidant coping strategies, where a higher score indicates greater inclination toward that coping mechanism. Most participants clustered within the moderate score ranges (1.51–2.5) across all three strategies, indicating moderate utilization of coping mechanisms overall.

Avoidant coping showed the highest concentration of participants in the 1.51–2.0 range, suggesting moderate reliance, with fewer participants exhibiting high avoidant scores. Problem-focused coping demonstrated a gradual increase, peaking in the 2.1–2.5 range, indicating stronger engagement with adaptive coping strategies among many participants. Emotion-focused coping showed a balanced distribution, with participants spread across moderate to higher score ranges. Very low (0–1) and very high (≥3.5) coping scores were comparatively uncommon. As the figure is descriptive, interpretations are limited to observed distributional trends rather than inferential conclusions.

### Statistical findings overview

Inferential analyses demonstrated that academic-related stress was the only stress domain showing significant variation across academic years, with final-year students reporting significantly higher levels than most other cohorts (Kruskal–Wallis H = 48.72, *p* < 0.001). Other stress domains did not differ significantly by academic year. Distributional analysis further revealed significant differences in stress severity across domains (χ^2^ = 364.60, *p* < 0.001), with academic stress contributing disproportionately to high and severe stress categories. Coping strategy preferences remained stable across academic years, with no significant differences observed in problem-focused, emotion-focused, or avoidant coping scores. Correlation analyses showed weak but significant positive associations between overall stress and all coping strategies, indicating that higher stress levels were associated with increased use of coping behaviors in general rather than selective reliance on adaptive strategies alone. Together, these findings identify academic stress as the principal variable influencing year-wise variation while suggesting relative stability in coping patterns across training stages (Table 4).

**Table 4:** Summary of inferential statistical analyses examining stress domains and coping mechanisms among MBBS students.

Analysis objective and statistical test	Variable/domain	Test statistic	*p*-value	Effect size	Interpretation
Year-wise comparison of stress domains Statistical Test- Kruskal– Wallis	Academic- related stress	H = 48.72	<0.001	ε^2^ = 0.146	Significant differences across academic years; highest stress in final year
	Interpersonal & intrapersonal stress	H = 3.84	0.428	–	No significant difference
	Teaching– learning stress	H = 4.42	0.352	–	
	Social- related stress	H = 2.21	0.697	–	
	Drive & desire stress	H = 2.77	0.598	–	
	Group activity stress	H = 7.53	0.110	–	
Post-hoc comparison (Academic stress) Statistical Test- Dunn– Bonferroni	Final year vs. First year	–	0.003	–	Final year significantly higher
	Final year vs. Second year	–	<0.001	–	
	Final year vs. Third year	–	<0.001	–	
	Second year vs. Third year	–	0.016	–	Second year higher
Distribution of stress severity across domains Statistical Test- Chi-square	Severity categories (mild–severe)	χ^2^ = 364.60 (df = 15)	<0.001	Cramer’s V = 0.255	Severity distribution differed across domains; academic stress showed higher severe burden.
Year-wise comparison of coping strategies Statistical Test- Kruskal– Wallis	Problem- focused coping	H = 5.27	0.261	–	No significant difference
	Emotion- focused coping	H = 4.97	0.291	–	
	Avoidant coping	H = 3.07	0.547	–	
Association between stress and coping Statistical Test- Spearman correlation	Stress vs. Problem- focused coping	ρ = 0.232	<0.001	–	Weak positive association
	Stress vs. Emotion- focused coping	ρ = 0.266	<0.001	–	
	Stress vs. Avoidant coping	ρ = 0.277	<0.001	–	

## Discussion

This study examined stress domains and coping strategies among MBBS students across academic years using validated instruments and inferential statistical analysis. The findings provide several important insights into the pattern and distribution of stress during undergraduate medical training.

The most notable finding was that academic-related stress was the only domain showing significant variation across academic years, with final-year students demonstrating significantly higher stress scores compared with junior cohorts. This finding suggests that progression through medical training contributes meaningfully to perceived academic burden and is consistent with earlier reports identifying examination pressure, increasing workload, and professional uncertainty as major stressors during advanced stages of medical education (1–3, 10, 11). The moderate effect size observed further supports the relevance of academic progression as an important contextual factor influencing stress levels among medical students (18).

In contrast, other stress domains—including interpersonal and intrapersonal stress, teaching–learning stress, social-related stress, and group activity-related stress—did not show statistically significant differences across academic years. These findings suggest that non-academic stressors may remain relatively stable throughout medical training, possibly reflecting consistent institutional environments, peer interaction patterns, and shared professional expectations across cohorts (4, 7). The absence of significant variation in these domains highlights the importance of distinguishing statistically supported findings from descriptive trends and underscores that not all perceived differences across training stages translate into measurable differences at the population level.

Analysis of coping mechanisms revealed that, although problem-focused coping had the highest overall mean scores, coping strategies did not significantly differ across academic years. Similar patterns of relatively stable coping styles across training stages have been reported in recent investigations of medical student coping behavior (19). This suggests that coping preferences may be established early during medical training and remain relatively stable as students progress through the curriculum. Furthermore, weak but significant positive correlations between overall stress and all coping categories indicate that increased stress is associated with greater use of coping behaviors in general rather than selective reliance on adaptive strategies alone. This nuanced pattern suggests that students respond to rising stress by mobilizing multiple coping responses simultaneously, including both adaptive and avoidant behaviors, a finding that aligns with contemporary perspectives describing coping as dynamic and multidimensional rather than strictly categorical (9, 13, 14). These findings highlight the complexity of stress–coping dynamics and suggest that coping responses may be influenced by factors beyond academic progression, such as personality traits, resilience, or social support systems.

The distributional analysis of stress severity demonstrated that academic-related stress contributed disproportionately to high and severe stress categories compared with other domains. This reinforces prior literature emphasizing academic performance pressure as a principal driver of psychological distress among medical students (1, 2, 6). At the same time, the predominance of mild stress levels in social and teaching–learning domains may indicate the presence of protective peer networks and institutional support structures, factors that have previously been associated with resilience and improved student well-being (13, 15). Recent meta-analytic evidence suggests that stress is strongly associated with burnout and psychological distress among medical trainees, highlighting the need for institutional attention to academic stressors (20).

### Strengths

The study has several strengths, including inclusion of participants from all academic years and internship, use of validated tools, and incorporation of inferential statistical analyses that permit interpretation beyond descriptive observations.

### Limitations

Nevertheless, certain limitations should be acknowledged. The cross-sectional design precludes causal inference and limits the ability to examine temporal changes in stress or coping. The reliance on self-reported data may introduce recall or social desirability bias, and the single-institution convenience sampling approach limits generalizability. In addition, because stress domains were assessed within the same participants, domain-wise comparisons should be interpreted as exploratory.

### Future directions

Collectively, the findings highlight academic stress as the principal domain exhibiting meaningful variability across medical training stages while suggesting relative stability in coping strategy patterns. The incorporation of inferential statistics strengthens confidence in these observations and provides a statistically grounded framework for future research. Longitudinal, multi-institutional studies incorporating objective psychological or physiological indicators may further clarify stress trajectories and the evolution of coping behaviors during medical education. The present findings should therefore be interpreted as exploratory but hypothesis-generating, offering empirical guidance for future investigations aimed at improving student well-being.

## Data Availability

The raw data supporting the conclusions of this article will be made available by the authors, without undue reservation.

## References

[1] Dyrbye LN Thomas MR Shanafelt TD. Medical student distress: causes, consequences, and proposed solutions. Mayo Clin Proc. (2005) 80:1613–22. doi: 10.4065/80.12.1613 16342655

[2] Hill MR Goicochea S Merlo LJ. In their own words: stressors facing medical students in the millennial generation. Med Educ Online. (2018) 23:1530558. doi: 10.1080/10872981.2018.1530558, 30286698 PMC6179084

[3] Rotenstein LS Ramos MA Torre M Segal JB Peluso MJ Guille C . Prevalence of depression, depressive symptoms, and suicidal ideation among medical students: a systematic review and meta-analysis. JAMA. (2016) 316:2214–36. doi: 10.1001/jama.2016.17324 27923088 PMC5613659

[4] Ishak W Nikravesh R Lederer S Perry R Ogunyemi D Bernstein C. Burnout in medical students: a systematic review. Clin Teach. (2013) 10:242–5. doi: 10.1111/tct.12014, 23834570

[5] Quek TT Tam WW Tran BX Zhang M Zhang Z Su-Hui Ho C . The global prevalence of anxiety among medical students: a meta-analysis. Int J Environ Res Public Health. (2019) 16:2735. doi: 10.3390/ijerph16152735 31370266 PMC6696211

[6] Frajerman A Morvan Y Krebs MO Gorwood P Chaumette B. Burnout in medical students before residency: a systematic review and meta-analysis. Eur Psychiatry. (2019) 55:36–42. doi: 10.1016/j.eurpsy.2018.08.006, 30384110

[7] Shanafelt TD West CP Sinsky C Trockel M Tutty M Satele DV . Changes in burnout and satisfaction with work-life integration in physicians. Mayo Clin Proc. (2019) 94:1681–94. doi: 10.1016/j.mayocp.2018.10.023 30803733

[8] Jackson ER Shanafelt TD Hasan O Satele DV Dyrbye LN. Burnout and alcohol abuse/dependence among US medical students. Acad Med. (2016) 91:1251–6. doi: 10.1097/ACM.0000000000001138, 26934693

[9] Kunwar D Risal A Koirala S. Study of depression, anxiety and stress among medical students in two medical colleges of Nepal. Kathmandu Univ Med J. (2016) 14:22–6. 27892436

[10] Kumar GS Jain A Hegde S. Prevalence of depression and its associated factors using Beck depression inventory among students of a medical college in Karnataka. Indian J Psychiatry. (2012) 54:223–6. doi: 10.4103/0019-5545.102412, 23226844 PMC3512357

[11] Abdulghani HM. Stress and depression among medical students: a cross-sectional study at a medical college in Saudi Arabia. Pak J Med Sci. (2008) 24:12–7.

[12] Hope V Henderson M. Medical student depression, anxiety and distress outside North America: a systematic review. Med Educ. (2014) 48:963–79. doi: 10.1111/medu.12512, 25200017

[13] Tempski P Santos IS Mayer FB Enns SC Perotta B Paro HB . Relationship among medical student resilience, educational environment and quality of life. PLoS One. (2015) 10:e0131535. doi: 10.1371/journal.pone.0131535, 26121357 PMC4486187

[14] Dyrbye LN Power DV Massie FS Eacker A Harper W Thomas MR . Factors associated with resilience to and recovery from burnout. Med Educ. (2010) 44:1010–22. doi: 10.1111/j.1365-2923.2010.03754.x 20880371

[15] Chew-Graham CA Rogers A Yassin N. ‘I wouldn't want it on my CV or their records’: medical students’ experiences of help-seeking for mental health problems. Med Educ. (2003) 37:873–80. doi: 10.1046/j.1365-2923.2003.01627.x, 12974841

[16] Yusoff MSB Abdul Rahim AF Yaacob MJ. The development and validity of the medical student stressor questionnaire (MSSQ). ASEAN J Psychiatry. (2010) 11:231–5.

[17] Carver CS. You want to measure coping but your protocol’s too long: consider the brief COPE. Int J Behav Med. (1997) 4:92–100. 16250744 10.1207/s15327558ijbm0401_6

[18] Jeyapalan T Blair E. The factors causing stress in medical students and their impact on academic outcomes: a narrative qualitative systematic review. Int J Med Stud. (2024) 12:195–203. doi: 10.5195/ijms.2024.2218

[19] Cummerow J Obst K Voltmer E Kötter T. Medical students’ coping with stress and its predictors: a cross-sectional study. Int J Med Educ. (2023) 14:11–8. doi: 10.5116/ijme.63de.3840, 36870063 PMC10693402

[20] Jahanaray M Pasha A Jahanaray A. Burnout and psychological distress across U.S. postgraduate trainees, fellows, and students: a comprehensive meta-analysis. J Am Coll Heal. (2026) 74:1–14. doi: 10.1080/07448481.2025.2611276 41604674

