# Correction: Academic stressors and coping strategies among MBBS students: a cross-sectional, year-wise comparative study

**DOI:** 10.3389/fmed.2026.1870431

**Published:** 2026-05-15

**Authors:** Priyanka Rathi, Keshav Banga, Himanshu Singh

**Affiliations:** National Institute of Medical Sciences and Research, Nims University Rajasthan, Jaipur, Rajasthan, India

**Keywords:** academic stress, coping behavior, cross-sectional studies, medical education, MSSQ

Affiliation “National Institute of Medical Sciences and Research, Nims University Rajasthan, Jaipur, Rajasthan, India” was erroneously given as “Department of Pharmacology, National Institute of Medical Sciences and Research, Jaipur, India.”

The funder “Nims University Rajasthan” was erroneously omitted. The correct **Funding** statement reads:

The author(s) declared that financial support was received for this work and/or its publication. This study received financial support from Nims University Rajasthan, Jaipur for the article processing charges (APC).

In the published article, the **Acknowledgments** section was erroneously omitted. An **Acknowledgments** section has now been added and reads:

The authors acknowledge Nims University Rajasthan, Jaipur for providing institutional support and a conducive research environment for this study.

The original version of this article has been updated.

